# Lectures on the Diseases of Infancy and Childhood

**Published:** 1854-06

**Authors:** 


*Lectures on the diseases of Infancy and Childhood.* By CHAS. WEST, M.D., Fellow of the Royal College of Physicians, &c., &c. Second American, from the Second and Enlarged London edition. Philadelphia, Blanchard and Lea, 1854.

THIS work has been in the hands of the profession since 1848, and has been received, in America at least, with very much favor. The diseases of infancy are of that class, in the treatment of which, the young physician finds the greatest embarrassment, To older patients he can address himself directly by spoken language and receive positive answers to his questions, but the little sufferer can appeal for aid only by sounds and signs, to which no definite meaning has been affixed; the accuracy of the diagnosis must in many instances depend on the proper interpretation of a hundred little circumstances in reference, most of which individually there is room for doubt. It is for this reason perhaps, that the warm-hearted sympathetic physician watches over the little sufferer with an intense anxiety, less frequently experienced in the treatment of adult cases. It is also for this reason that works devoted to infantile pathology and therapeutics, have been received with especial favor by the profession.

Of the work of Dr. West, it is unnecessary for us to speak in detail. The only objection perhaps that can be brought against it is, the almost constant recommendation to deplete; but this is not peculiar to our author, almost all of our writers on the diseases of children, tell us to bleed and leech, and cup and blister, and purge and vomit, to a far greater extent than most judicious practitioners are willing to follow.

Dr. West recommends the application of Argent Nit. in solution, to the faauces and larynx in cases of croup. It will be remembered that this was first suggested by Dr. Horace Green, of N. York. Dr. G. has recently published a small pamphlet entitled "Remarks on Croup aud its treatment," in which his views are presented and his mode of treatment illustrated, by reports of cases.

There is a natural tendency on the part of discoverers, to attach to the results of their labors an undue importance, and, we think it not improbable, that the value of Nitrate of Silver in the treatment of Croup and other affections of the throat and larynx, has been over estimated by Dr. G. It must be conceded however, that the profession are under lasting obligations to him, for calling their attention to a new use of this truly valuable agent of our Materia Medica. J.

For Sale by D. B. Cooke & Co., 138, Lake Street, Chicago.

